# Identification of the technical and medical requirements for HEMS avalanche rescue missions through a 15-year retrospective analysis in a HEMS in Switzerland: a necessary step for quality improvement

**DOI:** 10.1186/s13049-018-0520-3

**Published:** 2018-07-04

**Authors:** Alexandre Kottmann, Pierre-Nicolas Carron, Lorenz Theiler, Roland Albrecht, Mario Tissi, Mathieu Pasquier

**Affiliations:** 1 Emergency Department, Lausanne University Hospital, Lausanne, Switzerland; 2 Swiss Air Ambulance, Rega, Zürich, Switzerland; 3 Department of Anesthesiology and Pain Medicine, Inselspital, Bern University Hospital, Bern, Switzerland

**Keywords:** Avalanche, Rescue, Mission, HEMS, Technical rescue, Clinical exposure, Requirements, Quality improvement

## Abstract

**Background:**

Avalanche rescues mostly rely on helicopter emergency medical services (HEMS) and include technical rescue and complex medical situations under difficult conditions. The adequacy of avalanche victim management has been shown to be unexpectedly low, suggesting the need for quality improvement. We analyse the technical rescue and medical competency requirements of HEMS crewmembers for avalanche rescue missions, as well as their clinical exposure. The study aims to identify areas that should be the focus of future quality improvement efforts.

**Methods:**

This 15-year retrospective study of avalanche rescue by the Swiss HEMS Rega includes all missions where at least one patient had been caught by an avalanche, found within 24 h of the alarm being raised, and transported.

**Results:**

Our analyses included 422 missions (596 patients). Crews were frequently confronted with technical rescue aspects, including winching (29%) and patient location and extrication (48%), as well as multiple casualty accidents (32%). Forty-seven percent of the patients suffered potential or overt vital threat; 29% were in cardiac arrest. The on-site medical management of the victims required a large array of basic and advanced medical skills. Clinical exposure was low, as 56% of the physicians were involved in only one avalanche rescue mission over the study period.

**Conclusions:**

Our data provide a solid baseline measure and valuable starting point for improving our understanding of the challenges encountered during avalanche rescue missions. We further suggest QI interventions, that might be immediately useful for HEMS operating under similar settings. A coordinated approach using a consensus process to determine quality indicators and a minimal dataset for the specific setting of avalanche rescue would be the logical next step.

**Electronic supplementary material:**

The online version of this article (10.1186/s13049-018-0520-3) contains supplementary material, which is available to authorized users.

## Background

Avalanches account for about 100 registered deaths annually in the Alps, around 25 of which occur in Switzerland, and these numbers have remained stable over the last 20 years. [1] A large majority of the accidents occur during recreational activities in remote areas, and the rescue operations are mainly conducted by helicopter emergency medical services (HEMS).

Avalanche rescue missions are complex, often combining search and rescue aspects with medical emergencies that require specific medical knowledge and skills. [2] In particular, the management of avalanche victims in cardiac arrest (CA) is complex and has been the focus of most of the medical literature on avalanche accidents. Because CA in avalanche victims may have several aetiologies, rescuers must integrate numerous specific information to guide their management, and dedicated algorithms have been developed. [3, 4]

However, the compliance to these recommendations has recently been described as unexpectedly low. [4, 5] This may be partially explained by the difficult and hazardous environment in which avalanche rescues take place, and by the time pressure and the presumably low clinical exposure and experience of the rescuers. [5–8] Whatever the reasons, these findings suggest the need for quality improvement (QI) in avalanche rescue.

A dedicated checklist has been recently proposed to provide decision-making support during avalanche rescue, with the aim to enhance compliance with published algorithms and improve the management of avalanche victims. [3, 4, 9, 10] However, a more comprehensive and systematic QI approach has not yet been described for avalanche rescue. Baseline measurements are an essential first step of any QI approach, which help to identify potential problem areas. With this study, we therefore provide a baseline measurement through a retrospective analysis of 15 years of avalanche rescue by a Swiss HEMS. [11, 12] We aimed to describe the technical and medical requirements (search, rescue, and medical competencies) of crewmembers. We also evaluated their exposure to avalanche rescue missions and victims. Finally, the goal of this study is to identify areas that should be the focus of future QI efforts.

## Methods

### Study design and population

We screened all HEMS primary missions from Rega-Swiss Air Ambulance between January 2001 and May 2016. [13] We included data from all patients caught by an avalanche (regardless of the burial degree), reached within 24 h of the alarm being raised, and transported by the HEMS. Individuals who were not caught by an avalanche were excluded (e.g. bystanders).

The Swiss hospital network contains 12 level-1 trauma centres, eight of which have extracorporeal life support capabilities. Rega-Swiss Air Ambulance is the major HEMS in Switzerland and has 13 bases distributed along the Alps and near to the five university hospitals in the country. Every base is equipped with a single helicopter dedicated to HEMS missions and continuously available 24/7. The standard crew includes a pilot, a paramedic, and an emergency physician. As for mountain rescue missions, a specialist from the Swiss Alpine Rescue may be added to the standard crew. In the specific case of avalanche rescue missions, the HEMS helicopter is however most of the time dispatched directly to the avalanche site to minimize the response time, and an additional helicopter from a commercial company is dispatched simultaneously to pick up an incident commander, mountain rescue specialists and avalanche dogs if required.

### Database, variables, and definitions

Data were retrospectively analysed from the digital dataset of Rega-Swiss Air Ambulance. [14] The dataset is prospectively registered for resource management and activity documentation purposes after every mission, by the pilot for the operational part and by the physician or paramedic for the medical part. The operational data included the month and year of the avalanche accident, the response time (defined as the time interval between the alarm and the arrival of the first HEMS helicopter on site), the method used to evacuate the patient from the avalanche site, the patient’s destination, and the identification number of the crewmembers involved.

Medical data included the age and gender of the patient, the pre-hospital presumptive diagnoses made by the HEMS physician, and the diagnostic and therapeutic measures applied on site or during transport. Hypothermia is an item in the predefined list of presumptive diagnoses of the database and is either determined clinically by using the Swiss staging system, or by measuring a core temperature < 35 °C with an esophageal probe, which is the only available device in our HEMS. [15] The severity of the patient’s condition was graded using the eight-level pre-hospital NACA severity score, which is assigned by the HEMS physician at the end of the rescue mission (Additional file 1). [16, 17]

Text fields of the data set were searched for the following avalanche-specific important information: burial degree, burial time, and information on the patient’s location and extrication. Complete burial was defined as the burial of at least the head and chest. [3] We defined multiple casualty accidents as those with more than one caught person. We defined summer avalanches as those occurring from June 1st to October 31st. [4, 18]

### Study aim

We aimed to describe the technical and medical requirements of avalanche rescue missions in terms of search, rescue, and medical competencies of the HEMS crewmembers, and to evaluate their clinical exposure to avalanche rescue missions and victims. Based on these baseline measurements, we aimed to identify and discuss operational and medical areas of avalanche rescue missions that should be the focus of QI efforts, and suggest potential QI strategies.

### Statistical analysis

The data retrieved from the database were exported to Stata version 14 (Stata Corporation, College Station, TX, USA). Descriptive statistics included frequencies, mean, and standard deviation (SD), or median, interquartile range (IQR), and range as appropriate. Groups were compared using Pearson’s Chi^2^ or Fisher’s exact tests, as appropriate. A bilateral *p*-value < 0.05 was considered to indicate a significant difference.

## Results

**Fig. 1:**
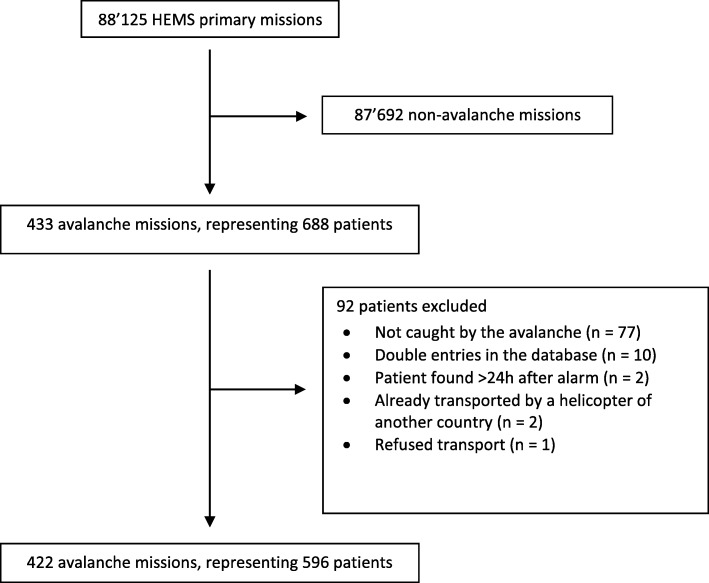
Flowchart of the study patients. Rega-Swiss Air Ambulance Helicopter Emergency Medical Service (HEMS) avalanche rescue missions between 1.01.2001 and 31.05.2016

A total of 422 avalanche missions (0.5% of the primary missions during the same period) were included in the study, representing 596 patients (Fig. 1). The mean annual number of avalanche missions was 27 ± 8. No significant differences in the number of rescues were detected between the years (*p* = 0.257). February was the busiest month, accounting for 27% (*n* = 113) of avalanche rescues. Only 2% (*n* = 7) were summer avalanche rescue missions. Avalanche accidents involved predominantly middle-aged males (80% men; mean age 39 ± 13 years).

The overall characteristics of the avalanche accidents are shown in Table 1. Box plots of the results presented as means, IQR and range are available in the Additional file 2**.**

**Table 1:** Overall characteristics of the 422 avalanche accidents involving 596 victims (Rega-Swiss Air Ambulance Helicopter Emergency Medical Service (HEMS), 1.01.2001 to 31.05.2016)

Search and rescue characteristics	
Avalanche accidents requiring a winch operation, n (%)	121 (29)
Avalanche accidents with at least one victim located by professional rescuers, n (%) ^a^	38 (49)
Avalanche accidents with at least one victim extricated from the avalanche by professional rescuers, n (%) ^b^	34 (31)
Avalanche victims (*n* = 596)	
Involved persons per avalanche accident, median (IQR; range)	1 (1–2; 0–15)
Injured persons per avalanche accident, median (IQR; range)	1 (1–1; 0–7)
Completely buried victims per avalanche accident, median (IQR; range) ^c^	1 (1–1; 0–5)
Avalanche accidents with multiple casualties, n (%) ^d^	134 (32)
Avalanche accidents with multiple completely buried casualties, n (%) ^c,e^	37 (14)
Cardio-respiratory arrest victims (n = 172)	
Avalanche accidents with one victim in cardiac arrest, n (%)	121 (29)
Avalanche accidents with multiple victims in cardiac arrest, n (%)	20 (5)

^a^Reported for 102 patients in 77 avalanche accidents. Professional rescuers: HEMS crew (*n* = 27 victims), avalanche dog (*n* = 14 victims), mountain rescuers (e.g., by probing) (*n* = 5 victims)

^b^Reported for 156 patients in 110 avalanche accidents

^c^Information about burial degree was available for 346 individuals in 258 avalanche accidents. Of these, 261 (75%) were completely buried, 61 (18%) partially buried, and 24 (7%) not buried

^d^Defined as avalanche with more than one person caught

^e^Defined as avalanche with more than one completely buried person

Two-hundred-and-seven (80%) of the 258 avalanches for which information about burial degree was available involved at least one completely buried victim. The median burial time (available for only 37 completely buried victims) was 35 min (IQR 15–60; range 3–1′140). Thirty-three percent (*n* = 141) of the avalanches involved at least one patient in CA. The median number of victims in CA per avalanche accident was 0 (IQR 0–1; range 0–5).

The median response time was 22 min (IQR 17–33, range 1–737). The proportion of patients suffering potential or overt vital threat (i.e., NACA score > 3) was 47% (Table 2), and 29% of the patients encountered were in CA. Forty-seven percent of the patients suffered from presumed traumatic injuries, especially to the extremities or head, while 13% (*n* = 78) were considered uninjured by the HEMS physician.

**Table 2:** Presumptive diagnoses, monitoring, medical procedures, treatments, and destination of the 515 injured avalanche victims according to their severity. Counts and percentages are expressed by patients. Rega-Swiss Air Ambulance Helicopter Emergency Medical Service (HEMS) avalanche rescue missions between 1.01.2001 and 31.05.2016. *P*-values derive from the Pearson’s Chi^2^ calculations, when comparing the difference between the different NACA score categories

	overall	NACA severity score					*P* value
		1–3	4	5	6	7	
Injured patients, n (%) ^a^	515	239 (40)	84 (14)	20 (3)	54 (9)	118 (20)	
Distribution of the patients with a given presumptive diagnosis in the different NACA score categories							
	n (%) percentages are expressed by row						
Trauma	**278 (54)**	172 (62)	62 (22)	8 (3)	7 (3)	29 (10)	< 0.001
Head	**80 (16)**	33 (41)	18 (23)	5 (6)	5 (6)	19 (24)	
Spinal	**48 (9)**	17 (35)	16 (33)	2 (4)	0 (0)	13 (27)	
Thoracic	**49 (10)**	17 (35)	18 (37)	1 (2)	4 (8)	9 (18)	
Abdominal	**14 (3)**	5 (36)	3 (21)	1 (7)	2 (14)	3 (21)	
Pelvic	**31 (6)**	11 (35)	12 (39)	1 (3)	1 (3)	6 (19)	
Femur	**50 (10)**	27 (54)	16 (32)	2 (4)	0 (0)	5 (10)	
Extremity (except femur)	**142 (28)**	105 (74)	33 (23)	2 (1)	0 (0)	2 (1)	
Hypothermia	**186 (36)**	75 (40)	47 (25)	17 (9)	27 (15)	20 (11)	< 0.001
Asphyxia	**102 (20)**	2 (2)	3 (3)	3 (3)	26 (25)	68 (67)	< 0.001
Miscellaneous ^b^	**16 (3)**	7 (44)	2 (13)	1 (6)	2 (13)	4 (25)	0.973
Patients of each NACA score category for whom the given monitoring, procedure or treatment was done							
	n (%), percentages are expressed by column						
Monitoring	**194 (38)**						
Pulse oximetry	123 (24)	34 (14)	47 (56)	12 (60)	25 (46)	5 (4)	< 0.001
ECG monitoring	103 (20)	2 (1)	12 (14)	5 (25)	37 (69)	47 (40)	< 0.001
Non-invasive blood pressure measurement	46 (9)	8 (3)	16 (19)	6 (30)	14 (26)	2 (2)	< 0.001
Oesophageal temperature measurement	39 (8)	4 (2)	1 (1)	1 (5)	17 (31)	16 (14)	< 0.001
End tidal CO_2_ measurement ^c^	25 (5)	0 (0)	0 (0)	0 (0)	21 (39)	4 (3)	< 0.001
Immobilisation, splinting, dressing	**173 (33)**						
Vacuum mattress	140 (27)	48 (20)	49 (58)	13 (65)	25 (46)	5 (4)	< 0.001
Cervical collar	57 (11)	17 (7)	18 (21)	7 (35)	10 (19)	5 (4)	< 0.001
Peripheral splint	17 (3)	10 (4)	6 (7)	0 (0)	0 (0)	1 (1)	0.056
Dressing/haemostasis	16 (3)	7 (3)	8 (10)	1 (5)	0 (0)	0 (0)	0.002
Pelvic sling	1(0.2)	0 (0)	1 (1)	0 (0)	0 (0)	0 (0)	0.273
Procedures and treatments	**327 (55)**						
Oxygen administration (facemask or nasal cannula)	160 (31)	14 (6)	42 (50)	19 (95)	42 (78)	43 (36)	< 0.001
Peripheral venous catheter insertion	127 (25)	38 (16)	40 (48)	12 (60)	19 (35)	18 (15)	< 0.001
External rewarming (hot packs and/or electric blanket)	109 (21)	48 (20)	40 (48)	11 (55)	10 (19)	0 (0)	< 0.001
Chest compression ^d^	88 (17)	0 (0)	0 (0)	0 (0)	45 (83)	43 (36)	< 0.001
Orotracheal intubation	77 (15)	0 (0)	0 (0)	1 (5)	44 (81)	32 (27)	< 0.001
Ventilation ^e^	110 (21)	0 (0)	3 (4)	4 (20)	52 (96)	51 (43)	< 0.001
Defibrillation	21 (4)	0 (0)	0 (0)	0 (0)	14 (26)	7 (6)	< 0.001
Intraosseous access ^f^	18 (7)	0 (0)	1 (2)	0 (0)	11 (38)	6 (11)	< 0.001
Mechanical chest compression ^g^	13 (12)	0 (0)	0 (0)	0 (0)	10 (71)	3 (14)	< 0.001
Pneumothorax decompression	1(0.2)	0 (0)	0 (0)	0 (0)	0 (0)	1 (1)	0.498
Dislocation reduction or fracture realignment	7 (1)	0 (0)	3 (4)	1 (5)	1 (2)	2 (2)	0.074
Infusions ^h^	67 (12)	11 (5)	19 (23)	7 (35)	23 (43)	7 (6)	< 0.001
Pharmaceutical agents ^i^	125 (24)	24 (10)	38 (45)	7 (35)	30 (56)	26 (22)	< 0.001
Patients of each NACA score category who were transported to the given destination ^j^							
	n (%), percentages are expressed by column						
ECLS Trauma centre	61 (12)	7 (3)	22 (26)	7 (35)	24 (44)	n.r.	< 0.001
Non-ECLS Trauma centre	69 (13)	28 (12)	15 (18)	6 (30)	18 (33)	n.r.	< 0.001
Non-trauma centre hospital	272 (53)	166 (70)	47 (56)	7 (35)	12 (22)	n.r.	< 0.001

^a^NACA Scores were missing for three patients, none of which were in CA. Seventy-eight (13%) patients were uninjured (NACA score of 0)

^b^Miscellaneous: burns (*n* = 0/0/1/0/1), frostbite (*n* = 1/1/0/0/0), drowning (n = 0/0/0/1/3), exhaustion (*n* = 2/1/0/0/0), nervous/psychologic (*n* = 4/0/0/1/0)

^c^Continuous waveform end tidal capnography (EtCO_2_) was documented in 31% of the intubated patients. EtCO_2_ was more frequently used in patients with a NACA score of 6 compared to patients with a NACA score of 7 (48% vs. 9%, *p* = 0.001)

^d^Among patients with a NACA score of 6, two were in respiratory but not cardiac arrest. In a third patient, chest compression was made with a mechanical chest compression only

^e^Ventilation, including bag mask ventilation (with or without consecutive intubation) and manual or mechanical ventilation after intubation (no supraglotic airway device or surgical airway was used)

^f^Percentages are calculated taking into account that data were only available from 2009 onwards

^g^Percentages are calculated taking into account that data were only available from 2012 onwards. For four patients with a NACA score of 6, the mechanical chest compression device was used in standby mode only (i.e., installed on a patient with a sufficient circulation without compressing the chest)

^h^Next to crystalloids, colloids were given until 2014 (*n* = 1/1/1/2/0)

^i^Pharmaceutical agents: analgesic agents (*n* = 21/34/5/1/0), catecholamines (including epinephrine, norepinephrine and phenylephrine) (n = 0/0/1/23/26), antiemetics (*n* = 10/10/1/0/0), hypnotics, sedatives or neuroleptics (n = 2/8/4/3/0), neuromuscular blocking agents (n = 0/0/1/2/0), antiarrhythmics (n = 0/0/0/5/0), parasympathicolytics (n = 0/0/0/2/0), bronchodilators (n = 0/1/0/0/0), antidote (n = 0/0/0/1/0), tranexamic acid (given from June 2013) (n = 0/1/0/0/0), and other agents (n = 0/0/0/0/3)

^j^ECLS: extracorporeal life support. One-hundred-eighty-seven (31%) patients were not transported to a hospital: medical practice (*n* = 3); non-medical place (*n* = 172; 40% uninjured, 18% with NACA 1–3, 40% NACA 7); unknown destination (*n* = 12, including six dead victims). Uninjured patients were transported to a trauma centre (n = 1), non-ECLS & non-trauma centre hospital (*n* = 6), non-medical place (*n* = 69), or unknown destination (n = 2). Eight (1%) patients were transported by terrestrial rescue, accompanied by the HEMS physician

*n.r* not relevant

Monitoring of vital signs was used for 33% of the patients and significantly more often in CA patients (54% vs. 24%, *p* < 0,001). Immobilisation, splinting or dressing techniques were applied in 29% of the patients. Beside chest compression, intubation was the most often performed procedure in CA patients transported to a hospital. The most frequently used pharmacological agents were analgesic drugs for patients with vital signs and catecholamines for patients in CA.

Exposure of the different HEMS bases and crewmembers to avalanche rescue missions and victims are showed in Table 3. Sixty percent (*n* = 254) of the missions were covered by two HEMS bases located in the eastern part of the Swiss Alps in a region known to have a high avalanche activity. [19] The raw data of the clinical exposure analysis are available in the Additional file 3.

**Table 3:** Clinical exposure of each helicopter bases, physicians, and paramedics involved in the avalanche rescue missions. Rega-Swiss Air Ambulance Helicopter Emergency Medical Service (HEMS) avalanche rescue missions between 1.01.2001 and 31.12.2015

		Overall	Cardiac arrest victims
Clinical exposure to avalanche accidents and victims, median (IQR, range)			
Annual avalanche accidents per HEMS base ^a^		1.1 (0.5–2.5; 0.1–7.8)	–
Annual avalanche victims per HEMS base ^a^		1.5 (0.5–3.3;0.1–10.6)	0.5 (0.1–1.1;0–3.3)
Avalanche victims per physician over the study period ^b^		2 (1–4;1–18)	1 (0–1;0–10)
Avalanche victims per paramedic over the study period ^b^		4 (1–10.5;1–69)	1 (0–3;0–20)
Number of HEMS bases who intervened for an annual mean of: n (%) ^a^			
	0 mission	0	–
	< 1 mission/year	5 (38)	–
	1–4 missions/year	6 (46)	–
	5–8 missions/year	2 (15)	–
Number of HEMS bases who transported an annual mean of: n (%) ^a^			
	0 victim	0	2 (15)
	< 1 victim/ year	5 (38)	7 (54)
	1–4 victims/year	5 (38)	4 (31)
	5–11 victims/year	3 (23)	0
During the study period, number of HEMS physicians who managed respectively: n (%) ^b^			
	0 victim	not available	90 (48)
	1 victim	89 (48)	61 (33)
	2–5 victims	66 (35)	32 (17)
	6–10 victims	22 (12)	3 (2)
	11–20 victims	9 (5)	0
During the study period, number of HEMS paramedics who managed respectively: n (%) ^b^			
	0 victim	not available	22 (34)
	1 victim	18 (28)	15 (23)
	2–5 victims	20 (31)	19 (30)
	6–10 victims	10 (16)	3 (5)
	11–20 victims	8 (13)	5 (8)
	21–30 victims	4 (6)	0
	31–60 victims	3 (5)	0
	61–90 victims	1 (2)	0

^a^We calculated the annual mean of avalanche accidents and victims for each of the 13 HEMS bases. The median, IQR and range of these means are presented

^b^Only the 186 physicians and 64 paramedics who had to manage at least one avalanche victim during the study period were included

## Discussion

To our knowledge, our study is the first to specifically analyse avalanche rescue using a QI approach (see below) and the largest among the available literature addressing the management of avalanche victims. Crews were frequently confronted with technical rescue aspects, such as winch operations, patient location and extrication, and multiple casualty accidents. Half of the patients suffered from a potential or overt vital threat, and one-third of patients were in CA. The medical management of the victims required a large array of basic and advanced medical skills. Clinical exposure to avalanche victims among the crew was low; almost half (48%) of victims were treated by a physician (28% for the paramedics) for whom that particular patient was their only avalanche victim during the 15-year study period.

### Technical requirements of HEMS avalanche rescue missions

Avalanche rescue missions frequently occur in terrain that is difficult to access and, therefore, winch operation was necessary to evacuate 29% (*n* = 121) of the patients. This confirms the feasibility of winching both medical competencies and patients representing medical emergencies. [8] Although this information was not specified in the database, we can presume that the physician may mostly have disembarked the helicopter while hovering, as winching was used for the patient and medical crew evacuation.

Burial time is considered as the most important prognostic factor for completely buried avalanche victims, whose survival drops from 91 to 34% as burial time increases from 18 to 35 min. [20–22] Thus, prompt companion rescue can increase the survival rate. [2, 21, 23, 24] Companion rescue is, however, not always successful. [21] Being often the first professional rescuers on scene, our crews were involved in locating and extricating patients in almost half of the avalanche accidents. Although we could not exclude an under-reporting of the extrication of victims by companions, this proportion is higher for our study than that reported by Mair et al., who found that only 12% of victims were still completely buried when the rescue teams arrived on scene. This may be explained by the shorter median response time in our study (22 min vs. 35 min for Mair et al.). [2] The response time found in our study also means that our crews arrived on-site during the very critical phase of the avalanche survival curve, during which survival drops dramatically, and during which timely extrication is crucial. [22] Our data, therefore, reinforce the importance of dispatching a HEMS helicopter directly to the avalanche scene, to rapidly deliver professional rescuers. [21, 25] These latter should be highly skilled at locating and digging out still completely buried victims, including in complex situations, such as when multiple casualties are completely buried, which was the case in 14% of the missions in our study.

### Medical requirements of HEMS avalanche rescue missions

The patients encountered by our crews showed a wide range of pathologies and severity. Despite our shorter response and burial times (35 min vs. 360 min described by Mair et al.), 29% of our patients were in CA, which is higher than reported by Mair et al. [26, 27] While the latter did not describe burial times for victims extricated by companions, they found a slightly higher rate of victims extricated by companions (62% vs. 59%), which might at least in part explain the different rates of patients in CA.

The management of completely buried avalanche victims in CA is complex and rescuers should follow the dedicated algorithm. [4] Cardiopulmonary resuscitation (CPR) was applied in 51% of the CA patients, whereas this value was 100% in the study by Mair et al. [2] In our study, all patients with a NACA score of 6 and in CA were transported to a hospital under CPR or after the return of spontaneous circulation. However, the low rate of CPR in patients declared dead on site (NACA 7) is worrisome, notably as previous publications have shown that at least some, but possibly a significant proportion of CA victims might have been declared dead on site without sufficient reason. [5, 28]

The patients encountered by our crews showed a wide range of pathologies and severity and required a large panel of procedures, including advanced procedures to be performed on site (e.g. tracheal intubation, pneumothorax decompression, administration of pharmacological agents over an i.v. or i.o. access). This confirms that advanced life support (ALS) is feasible at avalanche scenes, as recommended by international guidelines. [3, 4, 29, 30]

However, the low proportion of our patients who were monitored might appear suboptimal. This might be explained, at least partially, by its feasibility in difficult conditions and the low reliability of some of the equipment under very cold environments. Monitoring of any kind was more frequently used in CA patients, possibly because this is an essential part of their assessment and because of its relatively smaller benefit in less severely injured patients, in a system where a health care professional will constantly be beside the patient. [4, 31] The stress of the situation, as well as the cold environment might explain partially the surprisingly low rate of end-tidal CO_2_ (ETCO_2_) measurement for intubated patients. Although its monitoring was higher in patients with a NACA score of 6 than in patients with a NACA score of 7 (49% vs 9%) and was also used more systematically since 2010, EtCO_2_ should be monitored in every intubated patient, as it allows to confirm the tube placement and to evaluate the efficiency of CPR. Core temperature was available for only 19% of CA patients. This can be partially explained by the fact that its measurement is considered mandatory only for CA patients with long burial times. [3, 4]

As new technologies are being developed to improve the reliability of monitoring under cold environments, other devices have come up to facilitate the work of the rescuers. Among these, two have been introduced at our HEMS during recent years with a direct implication in the management of avalanche victims: (1) an intraosseous access device, which is a valuable and efficient alternative to intravenous access in cold environments, and therefore especially attractive in avalanche rescue and; (2) a mechanical chest compression device that has been shown not only to allow efficient CPR under difficult conditions, but also contributes to the safety of the rescuers by allowing them to quickly clear the avalanche scene with a patient in CA, or performing on flight CPR, as in the case of hypothermic avalanche victims. [7, 25, 32–35] Those two devices (and especially the mechanical chest compression device) have been used frequently since their introduction. This confirms the feasibility and attractiveness of their use in the setting of avalanche rescue missions.

In our study, only 27% of the injured patients were immobilized with a vacuum mattress, and 11% had a cervical collar. In a previous study, a spine fracture was found in 7% of avalanche victims and was confirmed to be the cause of death in 5.6% of cases. [30] This apparently low rate of immobilization could be partially explained by the application of evolving international guidelines, suggesting that rapid extrication, transportation and stabilisation of the patient should be prioritized over immobilization. [36, 37] Crewmembers should also rely on solid clinical experience, as “best practice” management must be balanced with the environmental context.

Finally, in accidents involving multiple casualties (32%) or multiple CA patients (5%), complex medical triage skills are required to allocate the available human, material, and transport resources to those patients with the greatest chance of survival. [38, 39]

### Clinical exposure to avalanche rescue missions and victims

We showed that our crews have extremely low clinical exposure to avalanche rescue missions and victims. To our knowledge, it is the first time that data on this are made available. This finding is important considering the variety of technical and medical skills that need to be applied under challenging conditions.

In our HEMS, physicians had a proportionally lower exposure than paramedics. As most of the paramedics spend 100% of their time with the HEMS and are assigned there for several years, most of the shifts are covered by physicians who are assigned there for a limited period (6–12 months), with the remainder of the shifts being covered by physicians with a part-time activity over several years (15 shifts/year to 50% activity rate).

The annual probability of the Rega physicians and paramedics to be involved in an avalanche rescue mission is proportional to their occupation rate and depends on the HEMS base where they are employed. The probability is higher in the HEMS bases located closer to the mountains (6 bases having 1–4 missions/year) than in the bases located near the big cities (5 bases having less than 1mission/year).

Although the probability is highest in the two HEMS bases that cover 60% of the avalanche accidents (and having 5–8 missions/year), the number of missions and managed patients remains low when distributed among the providers. However, our data is insufficient to analyse whether exposure influenced patient outcome.

### Identifying areas to focus on for quality improvement interventions

The data presented in this study allowed us to identify three areas (HEMS operations and technical rescue, medical management of avalanche victims, data collection for avalanche rescue missions) that should be the focus of QI efforts and to propose multifaceted interventions for HEMS operating avalanche rescue missions in similar settings (Table 4). [11, 12]

**Table 4:** Potential quality improvement areas, goals, and interventions in avalanche rescue

Area	Goals	Interventions	Target
HEMS operations, search & technical rescue	Optimize avalanche rescue readiness to improve time efficiency (reduce time to extrication) while maintaining or increasing safety	Checklist for daily control of the personal safety equipment (clothes, avalanche beacon, AvaLung)	Providers
		Checklist for daily control of the avalanche rescue material (Helicopter external avalanche beacon, RECCO® device, probes, shovels)	Providers
		Use of a standard operating procedure for avalanche rescue missions	Providers
		The HEMS physician systematically wears a harness for avalanche rescue missions	HEMS physician
		Regular training of winch procedures	Providers
	Achieve a high level of performance in locating and digging out avalanche victims to reduce time to extrication	Regular specific field training including single and multiple burial scenarios	Providers
Medical management	Improve quality level of avalanche victim management	Dissemination of guidelines, up-to-date algorithm and clinical practice recommendations at the beginning of the winter season	Providers
		Continuous audit of avalanche rescue missions and feedback to HEMS providers	Topic expert, project leader
		Standard operating procedure for the management of avalanche victims in cardiac arrest	Providers
		Targeted training delivered via workshops and field training at the beginning of the winter season	Providers
		Indoor medical management simulation	Providers
	Improve adequacy to the algorithm	Use of the Avalanche Victim Resuscitation Checklist	Providers
	Improve quality of information transmission on site and to the hospital teams	Use of the Avalanche Victim Resuscitation Checklist	Providers
Data collection	Improve quality & completeness of data in our database for avalanche rescue missions	Development of a template for uniform data documentation and reporting for avalanche rescue missions through a consensus process (minimal data set)	Expert panel
Continuous quality improvement	Measure and improve quality	Development of quality indicators for avalanche rescue missions and management of avalanche victims through a consensus process	Topic experts, providers, relevant stakeholders
		Monitoring of quality indicators & reporting to the crews	Quality manager

As a preliminary requirement, rescuers intervening on an avalanche scene should be comfortable in a mountainous environment and trained to work in difficult outdoor conditions.

The goal of any intervention in the field of operational and technical aspects of avalanche rescue is to improve time efficiency while maintaining or increasing safety. Considering that our response times correspond to the asphyxia phase, reducing them may directly and favorably influence time to extrication, and thus also patient outcome. Thus, it may be monitored as a quality indicator of avalanche rescue in the future.

On an organizational level, avalanche rescue readiness should be high, both from a material and individual point-of-view. Individual (e.g., clothes, avalanche beacon) and avalanche-specific search and rescue materials (e.g., helicopter’s external avalanche beacon, RECCO® device, probes, shovels) should be readily available and checked (e.g., through the use of a checklist, on a daily basis at the beginning of the shift), and their use incorporated into regular training by the crewmembers.

Additionally, standard operating procedures (SOPs) for avalanche rescue should be available, incorporated in training and used. Next to the mandatory personal safety equipment, we suggest that the physician should systematically wear the harness dedicated to winch operations when on avalanche rescue missions. These measures may improve time efficiency and increase safety by relieving the minds of the rescuers from organizational concerns and allow them to concentrate more on risk assessment and on the technical and medical aspects of the rescue. [6, 29]

On an individual level, specific training should be carried out to achieve a high level of performance in locating and digging out of avalanche victims, including single and multiple casualty scenarios. The crewmembers should also be regularly trained in winch procedures, to increase safety and efficiency.

Our data, as those of other studies, suggest potential improvements in the medical management of avalanche victims. [5, 28] Because of the low clinical exposure to avalanche victims and complexity and specificity of their management, we focused our initiatives on provider education and decision-making support. For example, guidelines and clinical practice recommendations (or even medical SOPs) could be disseminated to the providers at the beginning of the winter. Also, targeted practical training can be achieved through workshops and field training. [40] Additionally, indoor simulations for the management of avalanche victims, as well as the sharing with all the providers of the HEMS of experiences collected through a continuous audit of avalanche rescue missions may partially substitute the lack of experience. [41–44]

The use of the existing Avalanche Victim Resuscitation Checklist as a clinical decision support could improve adherence to the recommendations, as well as with information transmission on the scene and to the hospital teams. However, this has not been evaluated yet. [4, 9, 10, 25, 45]

Finally, research in the field of avalanche rescue and victim management is hindered by the low incidence of cases, as well as the heterogeneity, quality, and completeness of the collected datasets among the different HEMS. To address this concern, a template for uniform data documentation and reporting for avalanche rescue missions should be developed through consensus of experts. It would allow for better comparison of registries data and upcoming studies among different HEMS. [46]

To measure QI, as well as to provide feedback of clinical performance measurements to the providers and identify new areas that should be the focus of QI efforts, quality indicators for avalanche rescue and medical management of avalanche victims should be developed, monitored and reported prospectively as part of a continuous quality improvement program. [12]

### Limitations

Our study has some limitations. Firstly, the quality and completeness of some data reported may have suffered from the retrospective design of our study. Under-reporting of some variables in the database cannot be ruled out. This may especially be the case for some items that are not *must report* items, as are the avalanche specific information. The latter were reported on a voluntary basis by the providers into free text fields. This might account for several differences between our data and previous studies. [2, 21] Moreover, the overall reliability of the information regarding most of the items was high, and their completeness was about 100%.

Secondly, diagnoses were only presumptive, and classified in pre-defined categories. Nevertheless, completeness of presumptive diagnoses was high, as this was missing for only two patients with a NACA score ≥ 1. However, considering that diagnoses were made under time pressure, in a difficult environment and with fully dressed patients, significant injuries may have been overlooked, especially in severely injured patients (NACA score ≥ 4) as reported by Hasler & al. [27] Although greater completeness and precision of diagnoses would have been appreciated, this would have required the analysis of hospital or forensic data, which were not available. The absence of definitive diagnosis however did not impact the conclusions of our study, which was specifically quality-oriented.

## Conclusions

Our data demonstrates several critical points: avalanche rescue missions are rare events, and the clinical exposure for HEMS crewmembers is generally low. In our setting, primary dispatching of a HEMS helicopter allowed, in most cases, the HEMS crew to reach the scene during the critical “asphyxia phase” of the avalanche survival curve. Rescuers need to be prepared for search and extrication operations as well as rendering medical aid to patients in life threatening conditions caused by trauma, cold, asphyxia and combinations thereof.

The baseline measurements described here enabled us to describe the requirements for avalanche rescue missions in terms of technical needs and medical competencies, and to identify areas that should be the focus of QI efforts. Based on our findings, we suggested organisational, operational and individual QI interventions, that might be immediately useful for HEMS operating under similar settings. Further, the development and implementation of a template for uniform data reporting and documentation for avalanche rescue missions; and the development, monitoring and reporting of quality indicators for the management of avalanche victims are the logical next step.

## Additional files

Additional file 1: NACA score. (DOCX 48 kb)

Additional file 2: Box plots. (DOCX 349 kb)

Additional file 3: Clinical exposure raw data. (DOCX 62 kb)

## Abbreviations

ALS: Advanced Life Support
CA: Cardiac Arrest
CPR: Cardiopulmonary Resuscitation
EtCO2: End tidal CO_2_
HEMS: Helicopter Emergency Medical Service
i.o.: Intraosseous
i.v.: Intravenous
IQR: Interquartile Range
NACA: National Advisory Committee for Aeronautics
QI: Quality Improvement
Rega: REttungsflugwacht, Garde Aérienne
SD: Standard Deviation
SOP: Standard Operation Procedure
