# Supplementary material for: Identification of the technical and medical requirements for HEMS avalanche rescue missions through a 15-year retrospective analysis in a HEMS in Switzerland: a necessary step for quality improvement
Source: Scand J Trauma Resusc Emerg Med. 2018 Jul 4;26:54. doi: 10.1186/s13049-018-0520-3 (PMC6033290; doi:10.1186/s13049-018-0520-3)
Supplement: Supplementary file 1 — NACA score. (DOCX 48 kb) [file 13049_2018_520_MOESM1_ESM.docx]

**Supplementary file 1:** NACA Score

The severity scoring used to classify injury or illness severity at Rega - Swiss Air Ambulance, as originally described by the National Advisory Committee on Aeronautics (NACA).[1]

| NACA 0 | No injury or disease |
| --- | --- |
| NACA 1 | Injuries/diseases without any need for acute physicians care |
| NACA 2 | Injuries/diseases requiring examination and therapy by a physician, but hospital admission is not indicated |
| NACA 3 | Injuries/diseases without acute threat to life but requiring hospital admission |
| NACA 4 | Injuries/diseases that can possibly lead to deterioration of vital signs |
| NACA 5 | Injuries/diseases with acute threat to life |
| NACA 6 | Injuries/diseases transported after successful resuscitation of vital signs † |
| NACA 7 | Lethal injuries/diseases (with or without resuscitation attempts) † |

† The NACA severity score does not precise in which category a patient in CA transported under ongoing resuscitation (CPR) should be classified. In our study, we were able to confirm that all the patients with a NACA score of 6 and in cardiac arrest were transported under resuscitation (CPR) or after return of a spontaneous circulation and that all the patients with a NACA score of 7 were declared dead on site.

**REFERENCE**

1. Tryba MB, H.; Echtermeyer V.: **Klassifizierung von erkrankungen und verletzungen im notarztrettungssystemen.** *Notfallmedizin* 1980, **6:**725–727.
