# Supplementary material for: Identification of the technical and medical requirements for HEMS avalanche rescue missions through a 15-year retrospective analysis in a HEMS in Switzerland: a necessary step for quality improvement
Source: Scand J Trauma Resusc Emerg Med. 2018 Jul 4;26:54. doi: 10.1186/s13049-018-0520-3 (PMC6033290; doi:10.1186/s13049-018-0520-3)
Supplement: Supplementary file 2 — Box plots. (DOCX 349 kb) [file 13049_2018_520_MOESM2_ESM.docx]

**Supplementary file 2:** Box plots of the results presented as medians, IQR and ranges
