# Supplementary material for: Identification of the technical and medical requirements for HEMS avalanche rescue missions through a 15-year retrospective analysis in a HEMS in Switzerland: a necessary step for quality improvement
Source: Scand J Trauma Resusc Emerg Med. 2018 Jul 4;26:54. doi: 10.1186/s13049-018-0520-3 (PMC6033290; doi:10.1186/s13049-018-0520-3)
Supplement: Supplementary file 3 — Clinical exposure raw data. (DOCX 62 kb) [file 13049_2018_520_MOESM3_ESM.docx]

**Supplementary file 3 :** clinical exposure, raw data

1. **Clinical exposure of the HEMS bases**

|  | **Annual mean** | **Frequency**  **(number of bases)** |
| --- | --- | --- |
| **Avalanche accidents per HEMS base** |  |  |
|  | 0.07 | 2 |
|  | 0.13 | 1 |
|  | 0.47 | 1 |
|  | 0.73 | 1 |
|  | 1.07 | 1 |
|  | 1.13 | 1 |
|  | 2.07 | 1 |
|  | 2.13 | 1 |
|  | 2.53 | 1 |
|  | 3.80 | 1 |
|  | 5.53 | 1 |
|  | 7.73 | 1 |
|  | |  |
| **Avalanche victims per HEMS base** | |  |
|  | 0.13 | 2 |
|  | 0.07 | 1 |
|  | 0.53 | 1 |
|  | 0.80 | 1 |
|  | 1.33 | 1 |
|  | 1.47 | 1 |
|  | 2.60 | 1 |
|  | 2.73 | 1 |
|  | 3.27 | 1 |
|  | 5.13 | 1 |
|  | 6.93 | 1 |
|  | 10.60 | 1 |
|  | |  |
| **Avalanche victims in cardiac arrest per HEMS base** | |  |
|  | 0.00 | 2 |
|  | 0.07 | 1 |
|  | 0.13 | 1 |
|  | 0.27 | 1 |
|  | 0.33 | 1 |
|  | 0.47 | 1 |
|  | 0.73 | 1 |
|  | 0.80 | 1 |
|  | 1.07 | 1 |
|  | 1.20 | 1 |
|  | 2.13 | 1 |
|  | 3.27 | 1 |

1. **Clinical exposure of the included physicians**

|  | **Number of victims** | **Frequency**  **(number of physicians)** |
| --- | --- | --- |
| **Number of managed avalanche victims per physician during the study period** |  |  |
|  | 0 | Not available |
|  | 1 | 89 |
|  | 2 | 29 |
|  | 3 | 16 |
|  | 4 | 14 |
|  | 5 | 7 |
|  | 6 | 10 |
|  | 7 | 5 |
|  | 8 | 3 |
|  | 9 | 2 |
|  | 10 | 2 |
|  | 11 | 2 |
|  | 12 | 1 |
|  | 13 | 1 |
|  | 16 | 2 |
|  | 17 | 2 |
|  | 18 | 1 |
|  |  |  |
| **Number of managed avalanche victims in cardiac arrest per physician during the study period** |  |  |
|  | 0 | 90 |
|  | 1 | 61 |
|  | 2 | 18 |
|  | 3 | 9 |
|  | 4 | 4 |
|  | 5 | 1 |
|  | 6 | 2 |
|  | 10 | 1 |

1. **Clinical exposure of the included paramedics**

|  | **Number of victims** | **Frequency**  **(number of paramedics)** |
| --- | --- | --- |
| **Number of managed avalanche victims per paramedic during the study period** |  |  |
|  | 0 | Not available |
|  | 1 | 18 |
|  | 2 | 7 |
|  | 3 | 5 |
|  | 4 | 3 |
|  | 5 | 5 |
|  | 6 | 3 |
|  | 7 | 1 |
|  | 9 | 4 |
|  | 10 | 2 |
|  | 11 | 1 |
|  | 13 | 1 |
|  | 14 | 2 |
|  | 15 | 1 |
|  | 17 | 2 |
|  | 20 | 1 |
|  | 21 | 1 |
|  | 22 | 2 |
|  | 27 | 1 |
|  | 38 | 1 |
|  | 43 | 1 |
|  | 46 | 1 |
|  | 69 | 1 |
|  |  |  |
| **Number of managed avalanche victims in cardiac arrest per paramedic during the study period** |  |  |
|  | 0 | 22 |
|  | 1 | 15 |
|  | 2 | 6 |
|  | 3 | 6 |
|  | 4 | 3 |
|  | 5 | 4 |
|  | 6 | 2 |
|  | 7 | 1 |
|  | 11 | 1 |
|  | 12 | 1 |
|  | 14 | 2 |
|  | 20 | 1 |
